# Supplementary figures and images for: Formulation and evaluation of atorvastatin calcium trihydrate Form I tablets
Source: PLoS One. 2025 Feb 13;20(2):e0317407. doi: 10.1371/journal.pone.0317407 (PMC11825022; doi:10.1371/journal.pone.0317407)

## SUPPLEMENTARY FIGURES

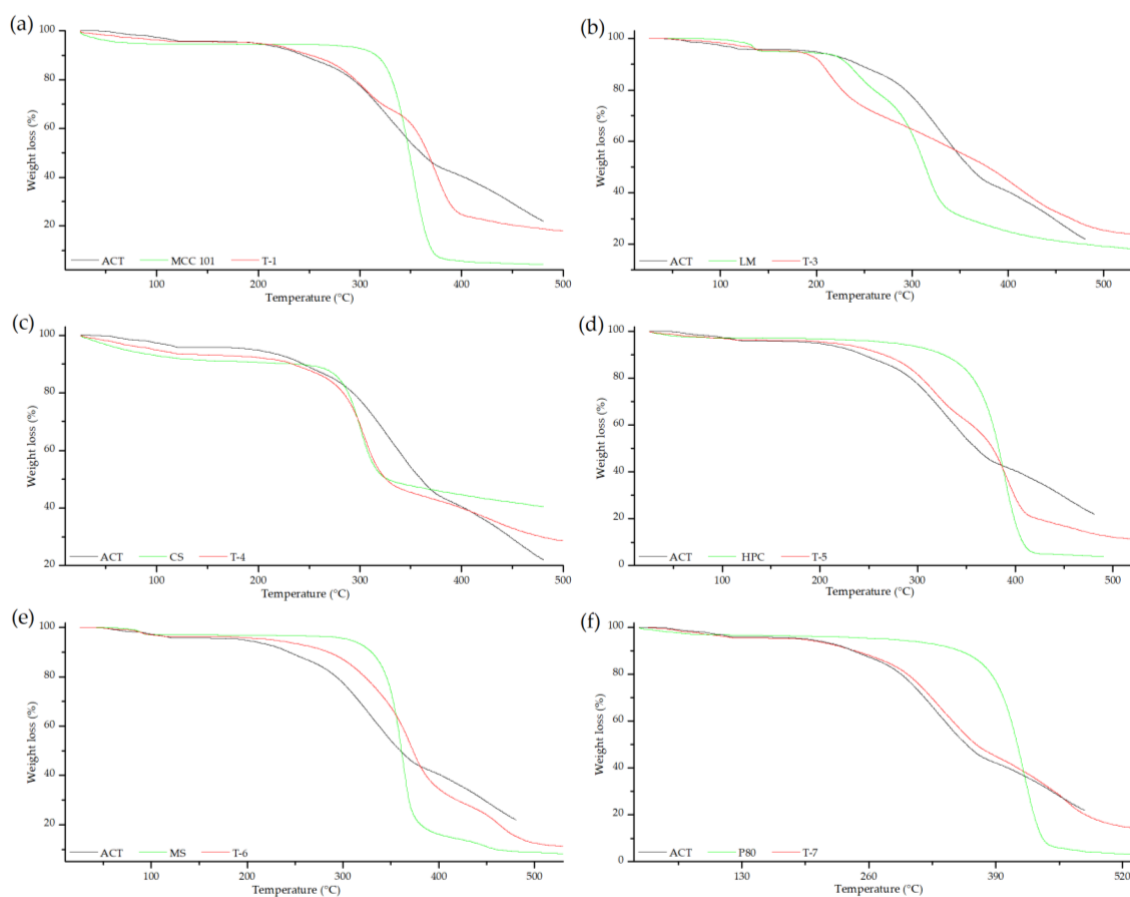

**S1 Fig. TGA curves of ACT, excipients, and mixtures:** (a) T-1, (b) T-3, (c) T-4, (d) T-5, (e) T-6, (f) T-7.

Supplement: S1 Fig — (PDF) [file pone.0317407.s001.pdf]
