# Supplementary material for: Formulation and evaluation of atorvastatin calcium trihydrate Form I tablets
Source: PLoS One. 2025 Feb 13;20(2):e0317407. doi: 10.1371/journal.pone.0317407 (PMC11825022; doi:10.1371/journal.pone.0317407)
Supplement: S2 Fig — (PDF) [file pone.0317407.s002.pdf]

SUPPLEMENTARY FIGURES

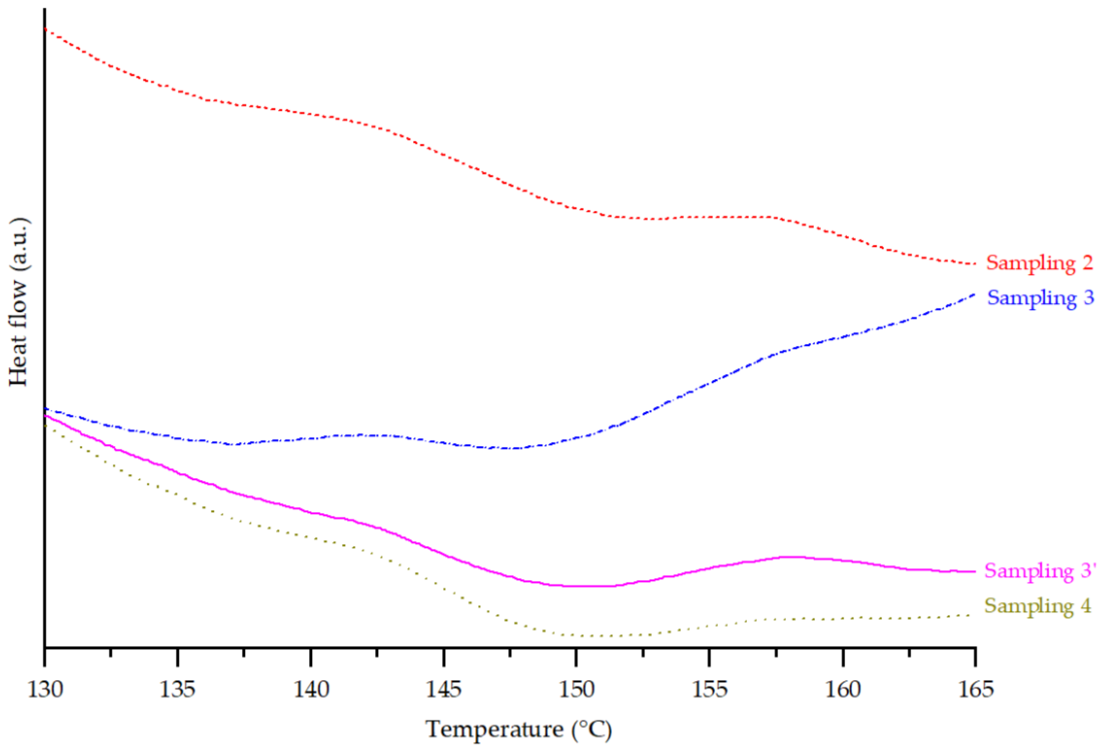

S2 Figure. DSC thermograms for ACT and samplings from the 40 mg tablet manufacturing process used to determine Tg of ACT.
