# Supplementary material for: Formulation and evaluation of atorvastatin calcium trihydrate Form I tablets
Source: PLoS One. 2025 Feb 13;20(2):e0317407. doi: 10.1371/journal.pone.0317407 (PMC11825022; doi:10.1371/journal.pone.0317407)
Supplement: Attachment — Submitted filename: Response to Reviewers-revAMAS.pdf [file pone.0317407.s003.pdf]

CeNAT

October 2nd, 2024

Re: Resubmission of manuscript PONE-D-24-20013

Thank you for the opportunity to revise and resubmit our manuscript titled “[Manuscript Title]” to [Journal Name]. We appreciate the time and effort that editor and the reviewer have dedicated to providing valuable feedback on our work. We have carefully considered all the comments and suggestions provided by the reviewers and have made the necessary revisions to improve the manuscript.

Below, we provide a detailed, point-by-point response to each of the editor and reviewer’s comments. We have addressed each point thoroughly and have indicated the changes made in the revised manuscript. We hope that these revisions meet the editor and reviewer’s expectations and enhance the quality of our paper.

Academic editor:

1. Please ensure that your manuscript meets PLOS ONE's style requirements, including those for file naming. The PLOS ONE style templates can be found at https://journals.plos.org/plosone/s/file?id=wjVg/PLOSOne_formatting_sample_main_body.pdf and https://journals.plos.org/plosone/s/file?id=ba62/PLOSOne_formatting_sample_title_authors_affiliations.pdf

The manuscript format has been modified and now meets PLOS ONE’s style requirements, including article title, corresponding authorship, levels 1 to 3 headings and file naming.

2. We note that the grant information you provided in the ‘Funding Information’ and ‘Financial Disclosure’ sections do not match. When you resubmit, please ensure that you provide the correct grant numbers for the awards you received for your study in the ‘Funding Information’ section.

Funding Information’ and ‘Financial Disclosure’ have been fixed. A grant number is not available (NA) for this funding. Since the National Center for High Technology of Costa Rica (CeNAT-CONARE) Scholarship Program was for the financial support awarded to the student during the research period the state "The funders had no role in study design, data collection and analysis, decision to publish, or preparation of the manuscript” has been included in the manuscript.

3. Thank you for stating the following financial disclosure: “CeNAT Scholarship Program”

Please state what role the funders took in the study. If the funders had no role, please state: "The funders had no role in study design, data collection and analysis, decision to publish, or preparation of the manuscript." If this statement is not correct you must amend it as needed. Please include this amended Role of Funder statement in your cover letter; we will change the online submission form on your behalf.

As mentioned in previous point, the Role of Funder was for the financial support awarded to the student during the research period. Therefore, the funders had no role in study design, data collection and analysis, decision to publish, or preparation of the manuscript. This state has been added in the cover letter.

CeNAT

4. In the online submission form, you indicated that "Data will be made available on request."

All PLOS journals now require all data underlying the findings described in their manuscript to be freely available to other researchers, either 1. In a public repository, 2. Within the manuscript itself, or 3. Uploaded as supplementary information. This policy applies to all data except where public deposition would breach compliance with the protocol approved by your research ethics board. If your data cannot be made publicly available for ethical or legal reasons (e.g., public availability would compromise patient privacy), please explain your reasons on resubmission and your exemption request will be escalated for approval.

We have revised the data and the manuscript and determined that relevant data are within the main manuscript and in the Supplementary material.

5. Please review your reference list to ensure that it is complete and correct. If you have cited papers that have been retracted, please include the rationale for doing so in the manuscript text, or remove these references and replace them with relevant current references. Any changes to the reference list should be mentioned in the rebuttal letter that accompanies your revised manuscript. If you need to cite a retracted article, indicate the article's retracted status in the References list and also include a citation and full reference for the retraction notice.

The reference list has been checked to ensure it is complete and correct. No changes had to be done.

Reviewer #1:

After reviewing the paper, I am pleased to report that I consider the article is well written and presents a solid piece of research. However, I have identified some areas that could be improved to further strengthen the quality of the document.

Force units

One of the main observations is that in some sections the International System (SI) is not used for force units. I suggest you review and update these units to conform to international standards. This will help improve the coherence and clarity of the manuscript.

Thank you for the observation. All force units were converted to International System (SI) units.

Use of the % sign

Additionally, I have noticed that in some cases the % sign is not separated from the number by a single space. I recommend that you review and correct these cases to maintain a consistent format throughout the text.

Thank you for pointing this out. The percentage sign (%) has been separated from the number by a single space throughout the manuscript.

Viewing the figures

On the other hand, I have observed that the difference between the dark colors in some of the figures could be further reinforced using different types of plotting. This would make it easier to view and understand the data

presented.

Thank you for your valuable observation. The figures have been adjusted accordingly. We hope that this modification meets the intention of making the data easy of viewing and understanding. However, if you have a specific suggestion for further improvement, we would greatly appreciate your input. In addition, we have marked the corresponding mixture and/or sampling in each figure.

Filter Information

I would also like to point out that when the use of filters is mentioned in the text, no information is given about the specific material of the filters used. This detail may be crucial for the reproducibility of future research based on this study. I suggest you include this information in the corresponding section.

Thanks for counting on me

Thank you for notices this. The information about the material and other specifications of the filters have been included in the corresponding sections.

Tel Central (506) 2519-5835, directo (506) 2519-5838
Apartado 1174-1200 Pavas, San José, Costa Rica.
aaraya@cenat.ac.cr
www.lanotec/cenat.ac.cr
