# Supplementary material for: Formulation and evaluation of atorvastatin calcium trihydrate Form I tablets
Source: PLoS One. 2025 Feb 13;20(2):e0317407. doi: 10.1371/journal.pone.0317407 (PMC11825022; doi:10.1371/journal.pone.0317407)
Supplement: Attachment — Submitted filename: Response to Reviewers-final.pdf [file pone.0317407.s005.pdf]

CeNAT

Journal Requirements:

Please review your reference list to ensure that it is complete and correct. If you have cited papers that have been retracted, please include the rationale for doing so in the manuscript text, or remove these references and replace them with relevant current references. Any changes to the reference list should be mentioned in the rebuttal letter that accompanies your revised manuscript. If you need to cite a retracted article, indicate the article's retracted status in the References list and also include a citation and full reference for the retraction notice.

The reference list has been checked to ensure it is complete and correct, without retracted papers. DOI corrections were made (References [32] and [51]).

Additional Editor Comments:

1. **Text style and format (e.g., P#6, L#117) is not uniform and needs to be checked and corrected throughout the manuscript**

The text style and format have been reviewed and corrected to ensure uniformity throughout the manuscript (P#3, L#67, P#6, L#121, 125, 129).

2. **P#6, L#129, There should be space between value and its unit (e.g., 10 mg), except percentage (10%). This point was also raised during previous round of the review. It needs to check and correct throughout the manuscript**

The space between values and their units has been corrected throughout the manuscript, under the guidance (P#6, L#137; P#13, L#269). For percentages, the space between the value and the '%' sign has been removed (P#7, L#146; P#13, L#270 and L#273; P#19, L#441; P#21, L#479 and L#480).

3. **Shift content of the section "Drug content Determination" with the Section "Assay" on P#6**

The content from the "Drug Content Determination" section has been shifted with the "Assay" section on P#6 and P#7.

4. **During compatibility study, was the effect of stress environmental conditions checked? Compatibility cannot be predicted from analysis of pure excipients and dosage form. Sample preparation is not in accordance with the reported literature like 10.1007/s11094-022-02556-8**

Thank you for your valuable feedback. We have addressed this point by including references to discuss compatibility studies, using accelerated conditions during preformulation, and support our

Tel Central (506) 2519-5835, directo (506) 2519-5838
Apartado 1174-1200 Pavas, San José, Costa Rica.
aaraya@cenat.ac.cr
www.lanotec/cenat.ac.cr

methodology (P#8, L#178-186, References [3,14,15]). Moreover, 10.1007/s11094-022-02556-8 was added as part of the discussion (Reference [13]).

5. **On P#13, it is stated that "Sampling points were chosen according to conditions considered critical known as process-induced transformations (PITs) for an API." Conditions critical for PITs need to be mentioned.**

The critical conditions for PITs have been specified as requested (P#14, L#295-297).

6. **General things e.g. P#14, L# 298 – 300, are added in "Results and Discussion" section which should be removed. Discussion should be precise to the results. Whole of the manuscript should be reviewed and general statements should be deleted.**

We recognize that general statements in the "Results and Discussion" section can shift the focus away from the findings. We carefully reviewed the manuscript and removed such statements, ensuring that the discussion remains directly tied to the results.

7. **Conclusion is too lengthy. It should be concise and based the experimental results**

The Conclusion has been changed to ensure it is clear and concise, emphasizing the main outcomes and their implications.

8. **In previous round of review, the reviewers suggested to evaluate the list of references which was not addressed, properly. Modify the list of references and add some relevant references like 10.1124/mol.117.110650, 10.1007/s11030-018-9839-y, 10.3390/toxins14120833 and 10.1109/TR.2023.3336330**

We have reviewed the reference list and confirmed that none of the cited papers have been retracted. All references have been verified for accuracy and completeness. DOI corrections were made for References [32] and [51].

Upon reviewing the suggested references, we carefully considered their relevance to the scope of our manuscript and incorporated those that aligned with the content of our work, such as 10.3390/toxins14120833, which has been added as Reference [7] (P#4, L#71-75).

The manuscript can be accepted for publication, subjected to the incorporation of suggested changes
