# Supplementary material for: Formulation and evaluation of atorvastatin calcium trihydrate Form I tablets
Source: PLoS One. 2025 Feb 13;20(2):e0317407. doi: 10.1371/journal.pone.0317407 (PMC11825022; doi:10.1371/journal.pone.0317407)
Supplement: Attachment — Submitted filename: Comments.docx [file pone.0317407.s004.docx]

**Comments**

- Text style and format (e.g., P#6, L#117) is not uniform and needs to be checked and corrected throughout the manuscript
- P#6, L#129, There should be space between value and its unit (e.g., 10 mg), except percentage (10%). This point was also raised during previous round of the review. It needs to check and correct throughout the manuscript
- Shift content of the section “Drug content Determination” with the Section “Assay” on P#6
- During compatibility study, was the effect of stress environmental conditions checked? Compatibility cannot be predicted from analysis of pure excipients and dosage form. Sample preparation is not in accordance with the reported literature like 0.1007/s11094-022-02556-8
- On P#13, it is stated that “Sampling points were chosen according to conditions considered critical known as process-induced transformations (PITs) for an API.” Conditions critical for PITs need to be mentioned.
- General things e.g. P#14, L# 298 – 300, are added in “Results and Discussion” section which should be removed. Discussion should be precise to the results. Whole of the manuscript should be reviewed and general statements should be deleted.
- Conclusion is too lengthy. It should be concise and based the experimental results
- In previous round of review, the reviewers suggested to evaluate the list of references which was not addressed, properly. Modify the list of references and add some relevant references like 10.1124/mol.117.110650, 10.1007/s11030-018-9839-y, [10.3390/toxins14120833](https://doi.org/10.3390/toxins14120833) and 10.1109/TR.2023.3336330

The manuscript can be accepted for publication, subjected to the incorporation of suggested changes
